# Advances in the Processing of UHMWPE-TiO_2_ to Manufacture Medical Prostheses via SPIF

**DOI:** 10.3390/polym11122022

**Published:** 2019-12-06

**Authors:** Rodrigo Ortiz-Hernández, Nicolás A. Ulloa-Castillo, José M. Diabb-Zavala, Alejandro Estrada-De La Vega, Jorge Islas-Urbano, Javier Villela-Castrejón, Alex Elías-Zúñiga

**Affiliations:** Departamento de Ingeniería Mecánica y Materiales Avanzados, Tecnologico de Monterrey, School of Engineering and Science. Av. E. Garza Sada 2501 Sur, Monterrey 64849, NL, Mexico; rortz.hdz@gmail.com (R.O.-H.); nicolas.ulloa@tec.mx (N.A.U.-C.); jdiabbzv@gmail.com (J.M.D.-Z.); alejandro_estrada@tec.mx (A.E.-D.L.V.); jor_isur@tec.mx (J.I.-U.); jvillela.cas@gmail.com (J.V.-C.)

**Keywords:** SPIF, nanocomposite, TiO_2_, nanoparticles, prosthetics, crystallization, UHMWPE

## Abstract

This research focuses on developing a novel ultra high molecular weight polyethylene (UHMWPE) material reinforced with titanium dioxide (TiO${}_{2}$) nanoparticles for producing craniofacial prostheses via an incremental sheet forming process (SPIF). First, UHMWPE-TiO${}_{2}$ nanocomposite sheets were produced using incipient wetting and the compression molding process by considering different concentrations of TiO${}_{2}$ nanoparticles. Then, the influence that the compression molding fabrication process has on the crystallinity and structural properties of the produced sample sheets was investigated. Experimental characterizations via scanning electron microscopy (SEM), differential scanning calorimetry (DSC), X-ray diffraction (XRD), Fourier transform infrared (FT-IR), tensile mechanical testing, and live/dead cell viability assays provided data that show an enhancement of the physical, mechanical, and biological properties. Finally, modifications on the nanocomposite material properties due to the SPIF manufacturing processes of a craniofacial prosthesis are addressed.

## 1. Introduction

In the last few decades, the investigations of advanced composite materials have become relevant for their potential use in biomedical applications [1]. To be specific, ultra-high molecular weight polyethylene (UHMWPE) composites have attracted attention for their mechanical performance [2], good thermal stability [3], and biocompatibility [4]—properties desirable for the development of biomedical devices. UHMWPE possesses long polymeric chains allowing the formation of extensive crystal domains and thus creating a polymeric material with a high crystallinity content [5], considered superior compared with other polymeric materials. The mechanical performance of UHMWPE can be improved by adding filler materials, nanoparticles (NPs), which act as a reinforcement in the polymeric structure. The selection of a filler material capable of increasing the mechanical properties and, at the same time, able to inhibit bacterial growth is of big relevance for biologic applications. TiO${}_{2}$ meets such requirements, it possesses an inert property and an inhibitor behavior that mitigates the proliferation of microorganisms [6]. In particular, the UHMWPE-TiO${}_{2}$ composite has been investigated for its improved mechanical properties [7,8,9], which are suitable for the manufacturing process to develop the geometry required in a zygomatic prosthesis. In this sense, the single point incremental forming (SPIF) manufacturing process has been used to develop geometries for prosthesis using polymers as polyvinyl chloride (PVC) [10], polyamide (PA) [11], polycarbonate (PC), and UHMWPE [12]. SPIF is a technique in which a material sheet (metal or polymer) is formed in a specific geometry by applying incremental deformations through a forming tool [13]. There is a progressively temperature-controlled deformation of the plastic integrity of the material sheet, in which the single point forming tool, guided by a CNC machine center tool path, applies a constant vertical and horizontal pressure on the surface of the material. The final geometry is reached when the material overpasses the elastic zone, and then it acquires the desired shape. Therefore, this research aims to manufacture by SPIF a UHMWPE-TiO${}_{2}$ nanocomposite sheet for the development of a functional zygomatic prosthesis.

In order to explore the degree of crystallinity and the influence of TiO${}_{2}$ NPs within the polymeric matrix, X-ray diffraction (XRD) and differential scanning calorimetry (DSC) were performed. The obtained results showed that the UHMWPE crystals reduced their symmetry from orthorhombic to the monoclinic phase during the elaboration of the sheet composites. The NP dispersion within the matrix was investigated by scanning electron microscopy (SEM), and Fourier transform infra-red (FT-IR) spectroscopy. Observe small agglomerations of NPs in the cross-sectional SEM images for those sheet composites with concentrations above 0.75%, while IR analyses detected the presence of carbon-oxygen vibrational modes which are related to the photocatalytic activity of TiO${}_{2}$ within the UHMWPE matrix. Tensile strength tests were carried out to evaluate the mechanical properties of the composite sheets, obtaining a better performance for the UHMWPE-TiO${}_{2}$ composite at 0.75%. Cell-adhesion and growth were performed in all UHMWPE-TiO${}_{2}$ composites validating their biocompatibility. The results reported in this work set the experimental parameters to develop a zygomatic prosthesis based on UHMWPE and TiO${}_{2}$ NPs.

## 2. Materials and Methods

The principal materials used in this study are Titanium (IV) oxide nanopowder (Anatase phase with an average particle size less than 25 nm) and UHMWPE (M${}_{w}$ 3,000,000–6,000,000), both purchased from Sigma-Aldrich (St. Louis, MO, USA). Isopropyl alcohol (2-Propanol alcohol, 99.5% purity grade) was bought from Jalmek Cientifica (Monterrey, México). The human fibroblast cell line (BJ, CRL-2522) was acquired from American Type Culture Collection (Manassas, VA, USA). Finally, the Dulbecco’s Modified Eagle Medium (DMEM/F12) supplemented with 10% fetal bovine serum was purchased from Gibco Invitrogen (Carlsbad, CA, USA). All the materials were used without any further purification.

### 2.1. Preparation of the UHMWPE-TiO${}_{2}$ Sheet Composite

The fabrication of the sheet nanocomposites was carried out in two stages. The first stage consisted of achieving an effective TiO${}_{2}$ NP dispersion in the UHMWPE polymeric matrix through an incipient wetting process. Therefore, the TiO${}_{2}$ NPs were initially dispersed in Isopropyl alcohol (100 mL) using an ultrasonic tip (30% of the maximum amplitude at 20 KHz) for 5 min. Then, UHMWPE dust (47 g) was incorporated into the dispersed NP solution and mixed in an ultrasonic bath (40 Hz) at room temperature for 10 min; at that time, the solution is mixed using a mechanical blender operated at 1500 rpm. Finally, the obtained solution was dried at 80 ${}^{\circ }$C for 6 h. The weight concentrations of the TiO${}_{2}$ NPs used to obtain each nanocomposite are described in Table 1.

**Figure 1:**
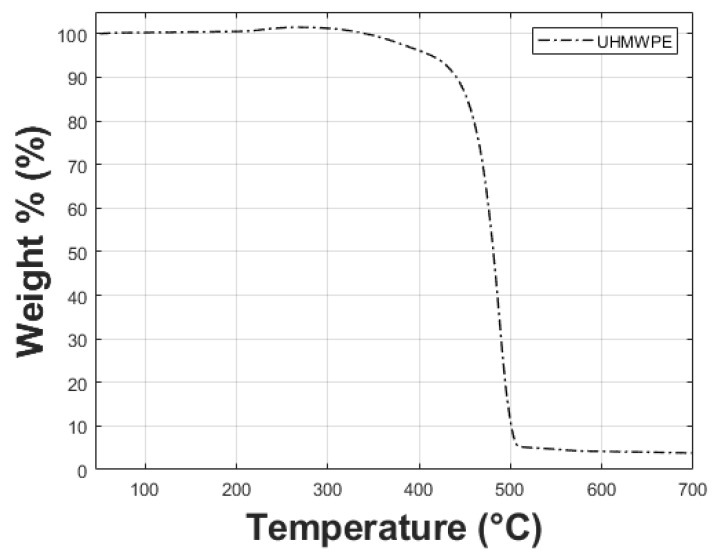
Thermogravimetric curve for neat UHMWPE. Notice that the material degradation temperature is above 400 ${}^{\circ }$C.

The manufacturing of the nanocomposite sheets was performed using a hot compression molding process. The obtained mixture, described in the previous procedure, was placed in a pre-heated mold (140 ${}^{\circ }$C) and pressed up to 22 metric tons in a Carver bench manual press (30 Ton capacity). Then, the temperature was increased up to 150 ${}^{\circ }$C (above the melting temperature) and maintained in such conditions for 25 min assuring an appropriate fabrication process. Thermogravimetric analysis (TGA) was performed on a neat UHMWPE material sample to assure that the fabrication temperature for the composite sheets is below the degradation temperature that is above 400 ${}^{\circ }$C, as observed in Figure 1. After the hot-pressing, the mold was cooled at 14 ${}^{\circ }$C and pressed up to eight metric tons for another 10 min. The dimensions of the obtained UHMWPE-TiO${}_{2}$ nanocomposite sheets were 150 mm × 150 mm × 2 mm. The procedure for the fabrication of the nanocomposite sheets is fully described in [14].

### 2.2. Geometry Formability of the Sheet Composites by SPIF

**Figure 2:**
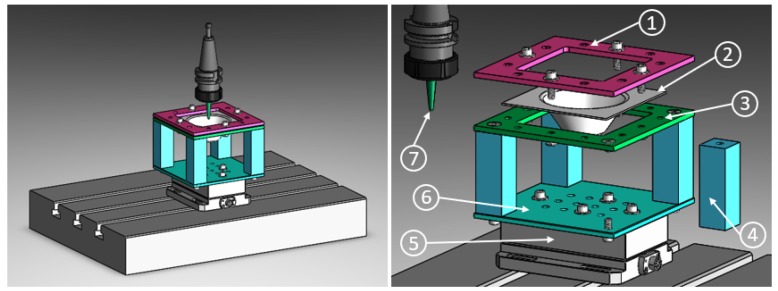
CNC milling machine set up for single point incremental forming process: (1) Top plate; (2) Sheet blank; (3) Clamping plate; (4) Hollow support; (5) Dynamometer; (6) Bottom plate and (7) Forming tool.

The UHMWPE-TiO${}_{2}$ nanocomposite sheets were formed by using a vertical CNC machine (Kryle VMC 535) which operates in the plastic regime. The experimental setup consists of a fixed square base with dimensions of 120 × 120 mm attached to a dynamometer (Kistler 9257B) as it is sketched in Figure 2. The path and geometry followed by the CNC machine are based in the model of an axi-symmetric part originated from the selection of an arc as a generatrix. The selected geometry allows the prediction of the wall thickness distribution along with the depth of the working sheet, as well as the calculation of the maximum elongation value reached during the forming process to avoid fractures [15]. The main experimental parameters controlled during the formability of the composite sheets and the values obtained for the generatrix tool path are displayed in Table 2. The formability of the sheet composites was carried out using a tool with a polished ball nose and mineral oil (6 mL) as a cutting lubricant. Additionally, the forces along the axis x,y, and *z* ($f_{x}$, $f_{y}$, and $f_{z}$, respectively), acting over the sheet composites during the conforming process were retrieved using a kistler piezoelectric dynamometer and a voltage amplifier as it is reported in [16].

### 2.3. Scanning Electron Microscopy (SEM)

The study of the surface morphology of the manufactured sheet composites was carried out by using a SEM (Zeiss model EVO MA 25) operated at 10.0 kV with a work distance of 7.0 mm.

### 2.4. Differential Scanning Calorimetry (DSC)

The degree of crystallinity and melting temperature (T${}_{m}$) were measured by using a DSC (PerkinElmer Pyris 8000). T${}_{m}$ was calculated considering the onset of the melting endotherm while the degree of crystallinity ($\chi_{c}^{dsc}$) was estimated using the enthalpy of melting change according to the equation: $\chi_{c}^{dsc}=\left[\Delta H_{m}/\Delta H_{m}^{\circ }\left(1-w_{t}\right)\right]\times 100$. Where $\Delta H_{m}$ and $\Delta H_{m}^{\circ }$ are the melting and the fully crystalline melting (289 J/g) enthalpies of the UHMWPE, respectively, and $w_{t}$ is the dopants weight fraction. All of the samples (average weight 10 mg) were held in standard aluminum pans and covers. The specimens were scanned from 30 ${}^{\circ }$C to 180 ${}^{\circ }$C with a heating increase rate of 10 ${}^{\circ }$C/min and using nitrogen as a purge gas. The DSC analysis curves for the material samples were carried out for the materials as received (first run data).

### 2.5. X-ray Diffraction Spectroscopy (XRD)

XRD measurements of the manufactured sheet composites, as well as for bare materials, were performed using a PanAnalytical (X’Pert Pro PW1800) diffractometer using Cu $K_{\alpha }$ radiation. The system was operated at 45 mA and 40 kV and the data was collected in the 2θ range of 10${}^{\circ }$–40${}^{\circ }$ with a scanning rate of 2${}^{\circ }$/min. The degree of crystallinity ($\chi_{c}^{xrd}$) was calculated using the equation: $\chi_{c}^{xrd}=\left[\sum I_{C}/\sum \left(I_{C}+I_{A}\right)\right]+\beta$; where $I_{C}$ and $I_{A}$ are the intensities for the crystalline and amorphous content, respectively, and β is the constant background (set by the experimental conditions during the measurements).

### 2.6. Fourier Transform Infrared Spectroscopy (FT-IR)

The chemical interaction between the TiO${}_{2}$ NPs and UHMWPE matrix was analyzed using a FT-IR (PerkinElmer Frontier) equipment with a UATR accessory. The procedure consisted of placing the developed sheet composites on the ZnSe-diamond crystal of the UATR and pressing with a tip to assure a good contact between the sample and the incident IR beam. The IR spectra were measured in the interval range of 4000 cm${}^{-1}$ to 400 cm${}^{-1}$ with a resolution of 8 cm${}^{-1}$, and considering an average of 16 scans. All measurements were performed by subtracting the baseline.

### 2.7. Tensile Strength Measurements

The mechanical integrity of the UHMWPE-TiO${}_{2}$ nanocomposites were inspected in a universal testing machine (Instron 3365), 5 kN load cell, with a crosshead speed of 50 mm/min${}^{-1}$ and in accordance with ASTM standard D638-14 for polymer type IV. The measurements were performed at room temperature and considering samples with dimensions of 33 mm × 6 mm × 2 mm.

### 2.8. Cell Culture Analysis (Adhesion/Viability)

The biological feasibility of the sheet composites was investigated by studying cell adhesion and cell growth, since it is desirable to quantify the ability of cells to attach to the UHMWPE-TiO${}_{2}$ nanocomposites. The human fibroblast cell line (BJ, ATCC CRL-2522™) was acquired from American Type Culture Collection (ATCC, Manassas, VA, USA) and maintained in Dulbecco’s Modified Eagle’s Medium/F12 (DMEM/F12) supplemented with 10% FBS at 37 ${}^{\circ }$C in a humidified atmosphere containing 5% CO${}_{2}$. In addition, Dulbecco’s modified Eagle’s medium/F12 (DMEM/F12), phosphate buffered saline (PBS), fetal bovine serum (FBS), penicillin-streptomycin, and trypsin were purchased from Gibco Invitrogen (Carlsbad, CA, USA). CellTiter96 AQueous one solution cell proliferation assays from Promega (Madison, WI, USA), and live/dead cell imaging kit from Molecular Probes, Life Technologies Corp (Carlsbad, CA, USA).

## 3. Results

### 3.1. Cross-Sectional SEM Analysis of the UHMWPE-TiO${}_{2}$ Sheet Composites

**Figure 3:**
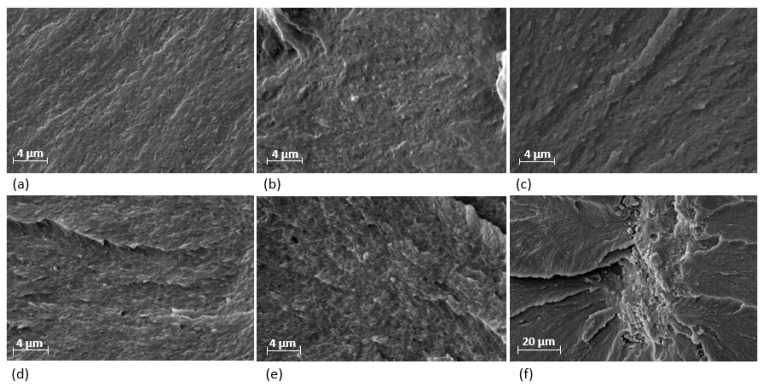
SEM images of the cross-sectional area for UHMWPE samples: (**a**) Reference, (**b**) M1, (**c**) M2; (**d**) M3; (**e**,**f**) M4. Satisfactory homogeneous dispersion of NPs are found at low concentrations (≤0.75 wt %) with considerable clusters after 1 wt %. No phase separations were found.

The morphology of the cross-sectional area of the sheet nanocomposites is shown in Figure 3. Despite the small agglomerations observed in samples M1, M2, and M3, the TiO${}_{2}$ NPs dispersion was satisfactory. The presence of such agglomerations is due to the difficulty of debinding some TiO${}_{2}$ NP clusters during the incipient wetting process. As it is reported in [7], for polymers composed of single carbon chains (PE and LDPE), the saturation level for TiO${}_{2}$ NPs occurs above 0.5%. However, the results in this work show a considerable increment of agglomerations in sample M4 (Figure 3e,f), which suggest that, for those polymers composed of long carbon-hydrogen chains (UHMWPE), the saturation level occurs above 1 wt %.

### 3.2. DSC and XRD

**Figure 4:**
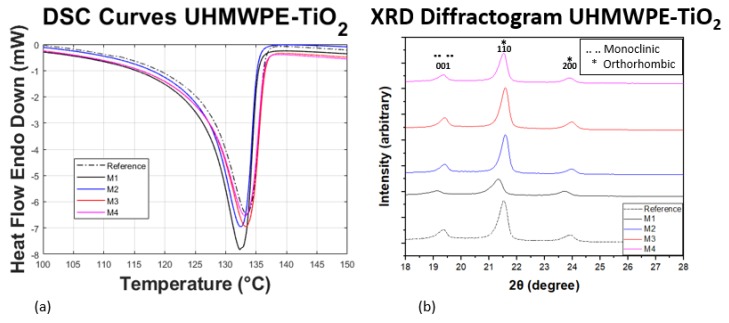
(**a**) DSC thermograms and (**b**) XRD diffractogram of the UHMWPE sample (Reference) and sheet composites (M1–M4). The degree of crystallinity behaves partially constant through the addition of TiO${}_{2}$ but with the formation of a monoclinic crystal phase as a consequence of the stress component induced during the hot-pressing manufacturing process.

The degree of crystallinity and structural information obtained from the composites were investigated by DSC and XRD; the results are shown in Figure 4. The values of T${}_{m}^{\mathrm{onset}}$, $\Delta H_{m}$ and the degree of crystallinity calculated by DSC ($\chi_{c}^{dsc}$) and XRD ($\chi_{c}^{xrd}$) are listed in Table 3. The endotherms for all the samples (Figure 4a) show similar behaviors, exhibiting a well defined line-shape between 110 ${}^{\circ }$C–138 ${}^{\circ }$C with an onset temperature for melting around 125 ${}^{\circ }$C. The relative increment of amplitude for M1–M4 indicates that the crystallization varies according to the filler contents [17]. This is confirmed by the slight shifts of the thermograms and diffractograms shown in Figure 4. The diffractograms for all samples show two peaks at 21.5${}^{\circ }$ and 23.9${}^{\circ }$, which correspond to (110) and (200) plane of an orthorhombic unit cell [18]; and an additional peak at 19.4${}^{\circ }$ corresponding to (001) plane of a monoclinic unit cell [18]. The presence of a well defined plane (001) at 19.4${}^{\circ }$ suggests that a stress component, induced during the hot-pressing manufacturing process, reduces a fraction of the symmetry from orthorhombic to monoclinic.

### 3.3. FT-IR

**Figure 5:**
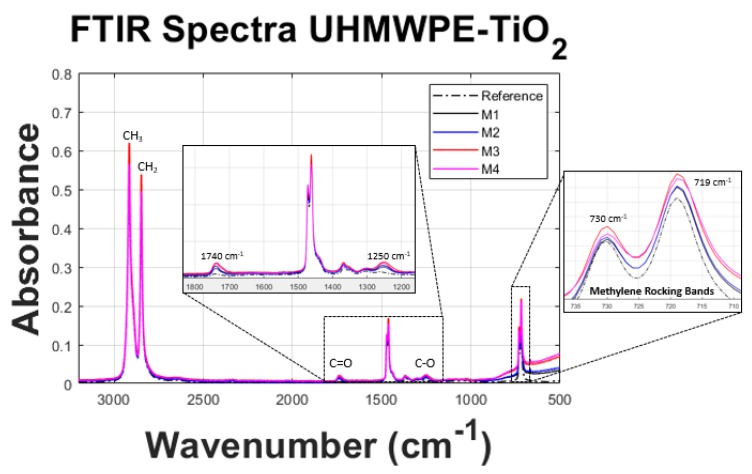
FT-IR spectra of the produced material samples. The well known vibrational bands corresponding to UHMWPE are visible in all the samples, with a positive interaction of TiO${}_{2}$ NPs with the polymeric chains, observable in bands 1740 cm${}^{-1}$ (C=O) and 1250 cm${}^{-1}$ (C-O).

**Figure 6:**
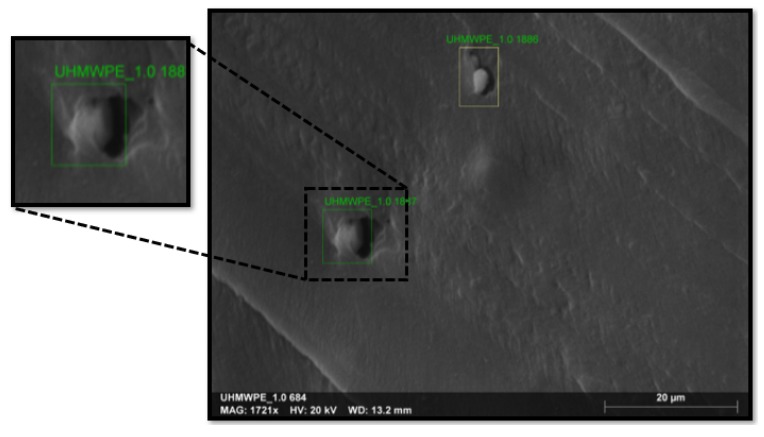
Energy dispersive X-Ray spectroscopy (EDS) micrograph that shows agglomerated TiO${}_{2}$ nanoparticles covered with UHMWPE.

The absorbance spectra for all the samples are shown in Figure 5. The results show that the position of infra-red absorption peaks of UHMWPE-TiO${}_{2}$ composites has no change with the addition of TiO${}_{2}$ NPs. The modes detected for UHMWPE Reference sample show peaks at 2924 cm${}^{-1}$ and 2851 cm${}^{-1}$, which correspond to the asymmetric and symmetric stretching mode of C-H, respectively. The mode at 1469 cm${}^{-1}$ represents the in-plane bending vibration of C-H, and the modes at 730 cm${}^{-1}$ and 719 cm${}^{-1}$ are related to methylene rocking vibrations, which are attributed to the high degree of polymerization and long molecular chains of UHMWPE [19]. The incorporation of the TiO${}_{2}$ NPs in the UHMWPE polymeric matrix promotes the carbon–oxygen interaction as it was detected in the modes at 1740 cm${}^{-1}$ (C=O) and 1250 cm${}^{-1}$ (C-O). The detection of such modes indicates that the TiO${}_{2}$ NPs are interacting with the polymeric chains thus the degradation of UHMWPE is caused by their photo-catalytic activity [20]. Additionally, as a result of the incorporation of TiO${}_{2}$ in different concentrations, the methylene rocking modes at 730 cm${}^{-1}$ and 719 cm${}^{-1}$ show an increment of amplitude which is associated with a crystallization process in the composite materials [21]. In summary, the carbon–oxygen interaction indicates that the TiO${}_{2}$ NPs are covered by polymeric chains. This is confirmed by the SEM-EDS image, Figure 6, that shows the presence of an amorphous layer of the polymer material that covers the TiO${}_{2}$ NP surface [9,22].

### 3.4. Mechanical Test Measurements

**Figure 7:**
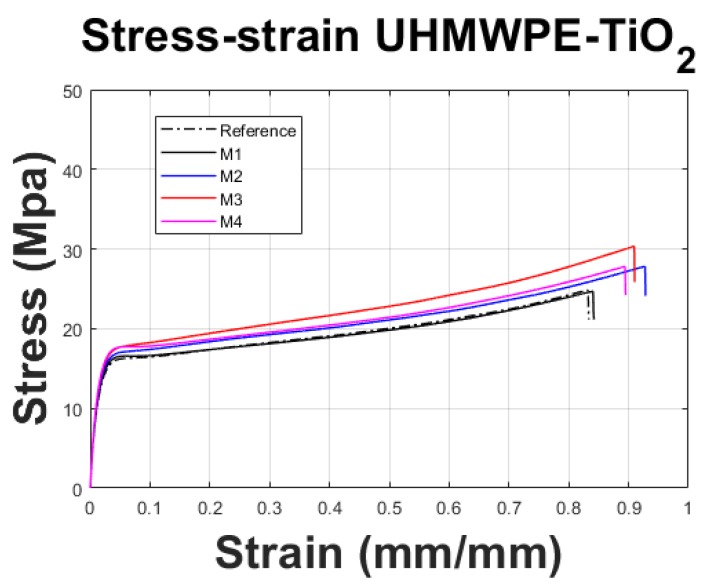
Stress–strain curves of UHMWPE-TiO${}_{2}$ nanocomposites. Mechanical properties increased with the addition of TiO${}_{2}$ NPs. For M3 sample, the UTS and strain elongation increased 30% and 10% respectively, before failure.

The results for the tensile strength measurements are illustrated in Figure 7, and the values retrieved from the testing are summarized in Table 4. As it is observed from the strain–stress curves, shown in Figure 7, there is an improvement in the tensile strength properties along with the increment of the TiO${}_{2}$ NPs concentration. The best tensile strength performance was achieved for the sample M3 having almost an increase in strain of 10% and of 30% in the ultimate tensile strength value when compared to the Reference material sample. This behavior is followed by a decay in the strain–stress response for sample M4. The mechanical properties observed in the composite sheet samples suggest that the maximum ultimate tensile strength value is achieved when a concentration of 0.75 wt % is added to the UHMWPE matrix. Furthermore, the maximum strain values of the reinforced samples, which are higher than that of the Reference, vary with the incorporation and concentration of TiO${}_{2}$ NPs, which influences the material composite ductility. The ultimate tensile strength (UTS) value is similar to the one reported by Efe et al. [17] in which a TiO${}_{2}$ concentration of 1 wt % was used. This improvement in our material response behavior could be due to the TiO${}_{2}$ NPs size (25 nm) used in our produced material samples that are half the size of those reported in [17].

### 3.5. Composite Sheets SPIF Process

**Figure 8:**
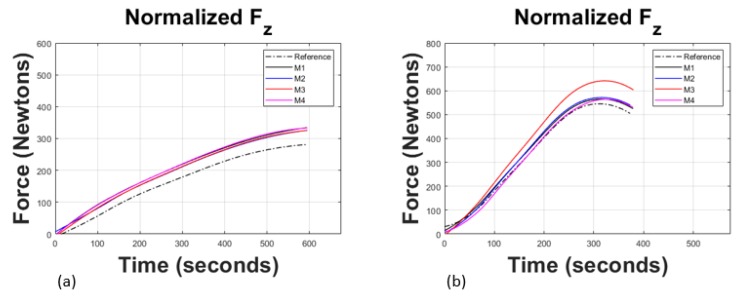
Normalized forces on the *z*-axis acting over the surface of the nanocomposite plate during SPIF: (**a**) with a forming tool diameter of 5 mm; (**b**) with a tool diameter of 10 mm. Notice that the magnitudes of the forming forces during the SPIF of the facial prostheses with a 5 mm tool diameter are lower than those recorded when using the tool with 10 mm.

The SPIF manufacturing process was carried out considering two different tool diameters, 5 mm and 10 mm, allowing for having a partial control in the deformation behavior of the UHMWPE-TiO${}_{2}$ composite sheets. The distribution curves of the forming forces along the deformation axis (f${}_{z}$) retrieved during the manufacturing process by SPIF are shown in Figure 8, and their maximum values are listed in Table 5. The forming forces experience a slight increase in their magnitude at increasing concentrations of TiO${}_{2}$ NPs. This tendency is also observed when a 10 mm tool diameter is used. However, when the concentration of the TiO${}_{2}$ is 1 wt %, the magnitude of the forming forces F${}_{z}$ decreases. This could be due to the agglomeration of the added NPs observed in Figure 3e,f, the reduction of their crystallization, as listed in Table 3, and to the TiO${}_{2}$ NP nucleation activity. The results show a similar qualitative behavior as those obtained by tensile strength tests shown in Figure 7. There, the highest ultimate tensile strength value is achieved by sample M3, confirming that the best mechanical performance of a material sample occurs for a TiO${}_{2}$ NP concentration of 0.75 wt %. The increment in the magnitude of the forming forces when a tool of 5 mm is used is because of the reduction of the contact area between the tool tip and the composite material samples [23].

**Figure 9:**
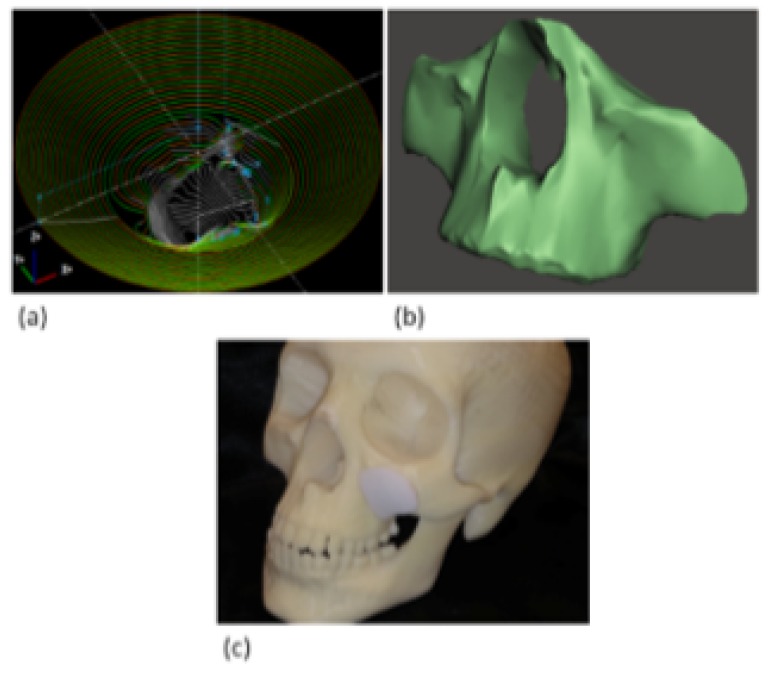
Facial prosthesis prototyping: (**a**) SPIF tool path followed by the CNC vertical milling machine for the fabrication of the prosthesis; (**b**) patient tomography; (**c**) manufactured facial prosthesis mounted on a human skull model.

Based on the above results, the craniofacial prosthesis was manufactured via SPIF by considering a 5 mm diameter tool in an attempt to produce a prosthesis with good geometrical accuracy. The dimensions of the prosthesis were retrieved from a tomography (imaging by sections) of a human skull (Figure 9b). The prosthesis was manufactured by considering an UHMWPE-TiO${}_{2}$ sheet nanocomposite for a TiO${}_{2}$ concentration of 0.75 wt %, which corresponds to the material sample with the best mechanical properties, and by considering the SPIF parameters listed in Table 2 for a tool of 5 mm. The produced UHMWPE-TiO${}_{2}$ prosthesis, mounted in the human skull model, is illustrated in Figure 9c.

### 3.6. Prosthesis, Cell Adhesion, and Cell Proliferation

Cells were seeded at 1 × 10${}^{4}$ cells/well and incubated at 37 ${}^{\circ }$C. After 4 h post-seeding, non-adherent cells were removed with a PBS wash, the remaining cells were detached with 0.25% trypsin/EDTA and the cell number was quantified according to the trypan blue exclusion assay. In addition, the results, expressed as a percentage of the original seeded cells, were also compared with a tissue culture treated plate.

To study cell proliferation, cells were seeded in a 96-well plate at a density of 1 × 10${}^{4}$ cells per well (3.12 × 10${}^{4}$ cells/cm${}^{2}$). Cell viability was evaluated after 24, 48, and 72 h post-seeding using CellTiter96 AQueous one solution cell proliferation assay. Then, 20 μL of CellTiter96 were added to each well and incubated at 37 ${}^{\circ }$C for 1 h; the supernatants were transferred to a new 96-well plate. The absorbance was acquired at 490 nm (Synergy HT, BioTec, Winooski, VT, USA). Each nanocomposite was compared against a tissue culture treated well (control) and the results were expressed as a percentage.

**Figure 10:**
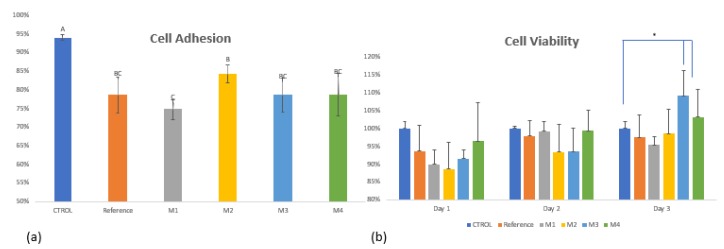
Fibroblast cell culture over the surface of UHMWPE (Reference) and UHMWPE-TiO${}_{2}$ sheet composites (M1-M4): (**a**) cell adhesion test after 4 h post-seeding for each sample; (**b**) cell viability after 24–48–72 h post-seeding. Appropriate adhesion of cells was observed after post seeding with a favorable environment for cell growth.

The biological feasibility of the sheet composites was investigated by studying cell adhesion and cell growth, and the results are shown in Figure 10. The percentage of adherent cells on the composite material samples was lower than the control well plate. Notice that the UHMWPE is able to support the cell attachment without any treatment due their biocompatibility properties. It is also observed in Figure 10 that the addition of up to 1 wt % of TiO${}_{2}$ has no statistical variations against UHMWPE; however, it has been reported that the use of higher TiO${}_{2}$ concentrations can increase the hydrophobicity and therefore improve cell behavior [24]. Furthermore, the results of cell viability through time, Figure 10b, indicates that all the produced samples do not have deleterious effects on cell growth. At the beginning of the assay, the cell viability of samples was among 88–95% compared with the control; after 72 h, the sample M3, with 0.75 wt % of TiO${}_{2}$, presented the highest viability value (109%). This shows that the addition of TiO${}_{2}$ to the polymeric matrix influences cells growth.

**Figure 11:**
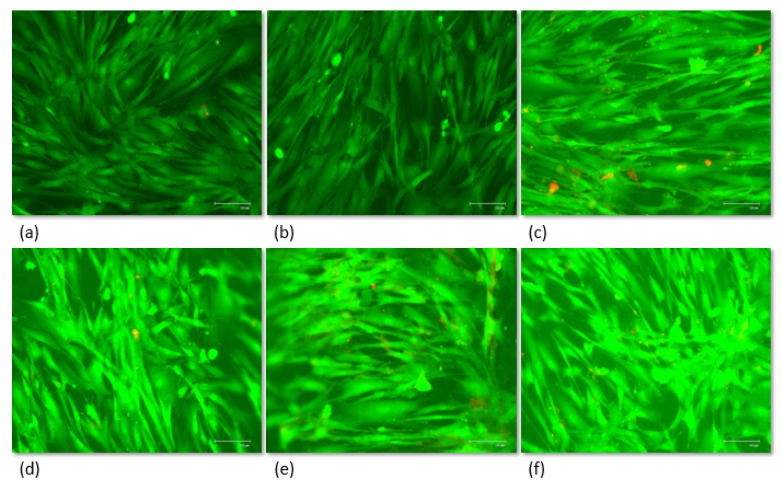
Fluorescence microscope images of human fibroblast cell growth on the surface of UHMWPE composites after 72 h: (**a**) control sample; (**b**) reference; (**c**) M1; (**d**) M2; (**e**) M3; (**f**) M4. Almost null cytotoxicity is reported.

The cell attachment was also observed by fluorescence microscopy (Figure 11). After 72 h post-seeding, cells were associated with a favorable growth due to the high confluence and uncompromised state (green color) and the almost null propidium iodine infiltration in treatments (red color). Moreover, the cells presented a normal elongated phenotype.

## 4. Conclusions

In this research work, UHMWPE-TiO${}_{2}$ nanocomposite sheets were manufactured by using incipient wetting and compression molding processes. Good dispersion of the TiO${}_{2}$ NPs was obtained in a liquid solution for low concentrations (≤0.75 wt %), as confirmed by SEM images. Experimental characterizations via DSC and XRD provided information that shows that the manufacturing compression molding process hinders the crystallization of the developed UHMWPE-TiO${}_{2}$ composite material because the forces induced during the hot-pressing manufacturing process tend to reduce a fraction of the symmetry from orthorhombic to monoclinic, which is confirmed by the thermograms and diffractograms slight shifts, illustrated in Figure 4. This implies that, although the degree of crystallinity was not increased during the development of the UHMWPE-TiO${}_{2}$ nanocomposites, the semi-crystalline arrangement in the polymeric chains was modified not only for the TiO${}_{2}$ NPs but also because of the stress induced in the material during the compression molding process. Furthermore, it was found that the ultimate tensile strength and strain deformation values of the produced nanocomposite sheets were superior to those of the Reference material sample reaching up to 10% of maximum strain before failure, and of improvement in the ultimate tensile strength value of about 30% for the concentration of 0.75 wt % of TiO${}_{2}$ NPs. These material properties are similar to those reported in [17] but with less wt % and half the size of TiO${}_{2}$ NPs. This increase in the stress values is reflected in higher forming forces of the UHMWPE-TiO${}_{2}$ nanocomposite sheets during the SPIF manufacturing of the craniofacial prosthesis. In fact, the forming forces needed to produce the prosthesis increase for higher concentrations of TiO${}_{2}$ NPs added to the polymeric matrix. However, when the concentration of the TiO${}_{2}$ NPs is 1 wt %, the magnitude of the forming forces decreases because of the agglomeration of the NPs and the reduction of the compound crystallization.

Finally, a fibroblast cells culture study was performed and monitored after 24, 48, and 72 h post seeding, finding almost null cytotoxicity and a favorable environment for cell proliferation.

## Figures and Tables

**Table 1:** Description of the amount of substances used for the preparation of the UHMWPE-TiO${}_{2}$ nanocomposites produced by incipient wetting and a hot compression molding process.

Sample	TiO_2_ (mg)	wt %
Reference	⋯	UHMWPE
M1	117.0	0.25%
M2	235.0	0.5%
M3	352.0	0.75%
M4	470.0	1.0%

**Table 2:** Parameters used during SPIF which allowed for controlling the wall thickness of the samples to avoid mechanical failures at its maximum deformation. Two scenarios were studied: (1) Tool diameter of 5 mm with a vertical step down of 0.25 mm; (2) Tool diameter of 10 mm with a vertical step down of 0.5 mm.

Parameters	SPIF	Generatrix Tool Path
Spindle speed	0 rpm	⋯
Tool diameter	5 mm and 10 mm	⋯
Feed rate	300 mm/min	⋯
Vertical step down (Δz)	0.25 mm and 0.5 mm	⋯
Initial diameter	⋯	100 mm
Generatrix radius	⋯	80 mm
Initial angle	⋯	45${}^{\circ }$
Exit angle	⋯	69.0${}^{\circ }$ and 78.0${}^{\circ }$
Maximum depth	⋯	28 mm and 40 mm

**Table 3:** Onset melting temperature (T${}_{m}^{\mathrm{onset}}$), enthalpy for melting ($\Delta H_{m}$) and degree of crystallinity ($\chi_{c}^{dsc}$,$\chi_{c}^{xrd}$) retrieved from DSC and XRD. The starting melting temperature or onset temperature is found around 125 ${}^{\circ }$C with a relative change in crystallinity originated from the contribution of TiO${}_{2}$ NPs and the crystallization of the composites during the hot-pressing manufacturing process.

Sample	T${}_{m}^{\mathrm{onset}}$ (°C)	Δ*H_m_* (J g^−1^)	$\chi_{c}^{\mathrm{dsc}}$ (%)	$\chi_{c}^{\mathrm{xrd}}$ (%)
Reference	126.0	91.0	31.5	31.9
M1	125.6	94.8	32.8	31.1
M2	125.7	92.8	32.3	30.4
M3	125.9	92.6	32.3	31.9
M4	125.9	88.6	30.9	29.2

**Table 4:** Material samples’ mechanical properties. The results obtained for the sample M3 exceed the ultimate tensile strength of all the samples and of the composite proposed in [17].

Sample	Average Yield Strength (MPa)	Average Ultimate Tensile Strength (MPa)	Average Young’s Modulus (MPa)	Average Maximum Strain (mm/mm)
Reference	15.1 ± 0.76	23.4 ± 0.53	450.5 ± 32.04	0.83 ± 0.05
M1	15.6 ± 0.83	24.7 ± 0.48	455.5 ± 14.0	0.84 ± 0.06
M2	15.9 ± 0.47	27.8 ± 2.23	476.0 ± 32.27	0.93 ± 0.042
M3	16.4 ± 0.14	30.4 ± 1.79	501.7 ± 16.71	0.91 ± 0.03
M4	16.6 ± 1.18	27.8 ± 1.48	509.3 ± 16.97	0.89 ± 0.095

**Table 5:** Maximum forces measured during SPIF with tool diameters of 5 and 10 mm.

Tool Diameter	5 mm	10 mm
Sample	F_z_ (N)	F_z_ (N)
Reference	299.59	577.66
M1	360.74	597.01
M2	345.42	602.38
M3	348.47	688.00
M4	351.52	595.33

## Abbreviations

The following abbreviations are used in this manuscript:

UHMWPE	Ultra High Molecular Weight Polyethylene
SPIF	Single Point Incremental Forming
